# Are current preventive chemotherapy strategies for controlling and eliminating neglected tropical diseases cost-effective?

**DOI:** 10.1136/bmjgh-2021-005456

**Published:** 2021-08-12

**Authors:** Hugo C Turner, Wilma A Stolk, Anthony W Solomon, Jonathan D King, Antonio Montresor, David H Molyneux, Jaspreet Toor

**Affiliations:** 1 MRC Centre for Global Infectious Disease Analysis, School of Public Health, Imperial College London, London, UK; 2 Oxford University Clinical Research Unit, Wellcome Africa Asia Programme, Ho Chi Minh City, Vietnam; 3 Centre for Tropical Medicine and Global Health, Nuffield Department of Medicine, University of Oxford, Oxford, UK; 4 Department of Public Health, Erasmus MC, University Medical Center Rotterdam, Rotterdam, The Netherlands; 5 Department of Control of Neglected Tropical Diseases, World Health Organization, Geneva, Switzerland; 6 Department of Tropical Disease Biology, Liverpool School of Tropical Medicine, Liverpool, UK; 7 Big Data Institute, Li Ka Shing Centre for Health Information and Discovery, University of Oxford, Oxford, UK

**Keywords:** health economics, onchocerciasis, soil-transmitted helminth infections, schistosomiasis, trachoma

## Abstract

Neglected tropical diseases (NTDs) remain a significant cause of morbidity and mortality in many low-income and middle-income countries. Several NTDs, namely lymphatic filariasis, onchocerciasis, schistosomiasis, soil-transmitted helminthiases (STH) and trachoma, are predominantly controlled by preventive chemotherapy (or mass drug administration), following recommendations set by the WHO. Over one billion people are now treated for NTDs with this strategy per year. However, further investment and increased domestic healthcare spending are urgently needed to continue these programmes. Consequently, it is vital that the cost-effectiveness of preventive chemotherapy is understood. We analyse the current estimates on the cost per disability-adjusted life year (DALY) of the preventive chemotherapy strategies predominantly used for these diseases and identify key evidence gaps that require further research. Overall, the reported estimates show that preventive chemotherapy is generally cost-effective, supporting WHO recommendations. More specifically, the cost per DALY averted estimates relating to community-wide preventive chemotherapy for lymphatic filariasis and onchocerciasis were particularly favourable when compared with other public health interventions. Cost per DALY averted estimates of school-based preventive chemotherapy for schistosomiasis and STH were also generally favourable but more variable. Notably, the broader socioeconomic benefits are likely not being fully captured by the DALYs averted metric. No estimates of cost per DALY averted relating to community-wide mass antibiotic treatment for trachoma were found, highlighting the need for further research. These findings are important for informing global health policy and support the need for continuing NTD control and elimination efforts.

**Summary box**

- Several of the most prevalent neglected tropical diseases (NTDs), namely lymphatic filariasis, onchocerciasis, schistosomiasis, soil-transmitted helminthiases and trachoma, are controlled at least in part by preventive chemotherapy (or mass drug administration).
- Many studies have found preventive chemotherapy to be a cost-effective strategy for controlling these NTDs.
- These findings have important implications for advocacy groups and potential funders and will be useful for decision-makers in endemic countries to justify the increased domestic healthcare spending needed for NTD programmes.
- Further work is needed to inform preventive chemotherapy programmes and the economics of elimination programmes, particularly for trachoma where no relevant estimates were found.

## Introduction

The neglected tropical diseases (NTDs) are a diverse group of conditions that are most prevalent in populations living in poverty.^1^ Several of the most prevalent NTDs, namely lymphatic filariasis, onchocerciasis, schistosomiasis, soil-transmitted helminthiases (STH) and trachoma, are controlled at least in part by preventive chemotherapy (also referred to as mass drug administration)^2^: the large-scale distribution of medicines to eligible populations within an endemic area, without diagnosing or testing individual participants for current infection.^3^ Some treatment programmes specifically target school-aged children (SAC), whereas others target the whole community.^3^ Within WHO guidelines, different preventive chemotherapy strategies may be recommended depending on prevalence of infection for these NTDs. Generally, there is a minimum prevalence below which mass treatment is not recommended.^3^

**Figure 1:**
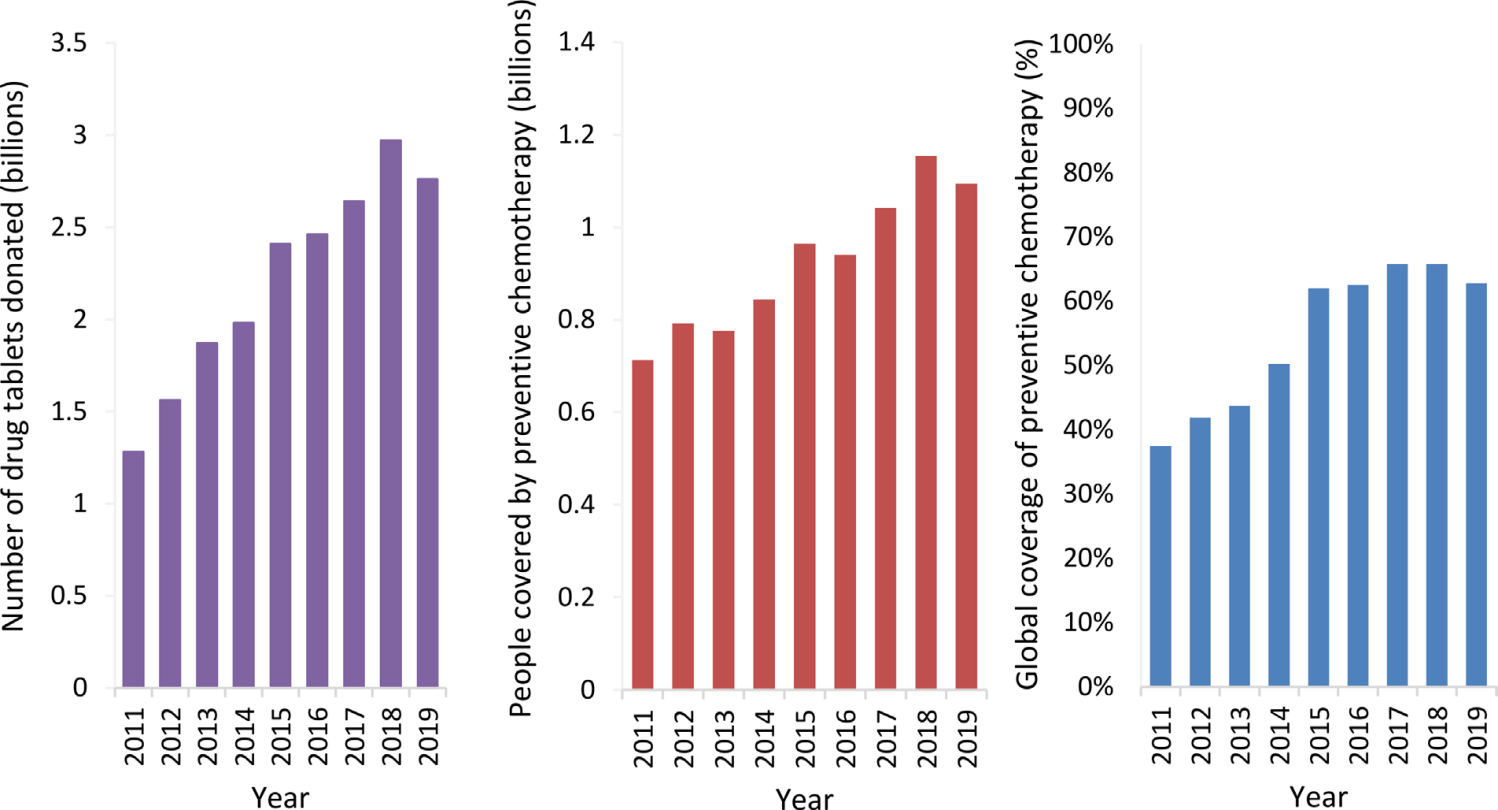
Summary of global preventive chemotherapy for lymphatic filariasis, onchocerciasis, schistosomiasis, soil-transmitted helminthiases and trachoma from 2011 to 2019. Data adapted from the WHO NTD progress dashboard.^100^ Global coverage is based on the proportion of the population requiring treatment that is treated.

When first used, preventive chemotherapy was often performed by mobile teams of paid health workers.^4^ However, in the mid-1990s, onchocerciasis programmes shifted to using community-directed distributors.^4–6^ Later other NTD programmes also started using community volunteers, and school-based preventive chemotherapy programmes incorporated teachers and other school officials as part of the NTD workforce.^3–9^ This resulted in a notable reduction in delivery costs and an increase in programmatic feasibility, allowing preventive chemotherapy programmes to expand. Over the last 20 years, the coverage of preventive chemotherapy has increased significantly (figure 1), supported by generous drug donations from the pharmaceutical industry (table 1).^10 11^ In 2019 alone, 1.63 billion preventive chemotherapy treatments were delivered for NTDs worldwide.^12^

**Table 1:** 2030 goals and drug donations for lymphatic filariasis, onchocerciasis, schistosomiasis, soil-transmitted helminthiases and trachoma

Disease	WHO 2030 goal^11^	Coverage achieved in 2019^12^	Drug donor and donation^11^
Lymphatic filariasis (elephantiasis)	Elimination as a public health problem (infection sustained below transmission assessment survey thresholds for at least 4 years after stopping mass drug administration; availability of essential package of care in all areas of known patients) validated in 58 (81%) countries	62.7%	Eisai: up to 400 million diethylcarbamazine (DEC) tablets per year until elimination Merck Sharp & Dohme: unlimited ivermectin in settings co-endemic with onchocerciasis (recently expanded to include up to 250 million tablets per year through 2025 for communities eligible for the triple-therapy mass drug administration regimen of ivermectin, DEC and albendazole) GlaxoSmithKline: up to 600 million albendazole tablets annually until elimination
Onchocerciasis (river blindness)	Elimination of transmission verified in 12 (31%) countries	62.8%	Merck Sharp & Dohme: ivermectin for as long as needed
Schistosomiasis (bilharzia)	Elimination as a public health problem (currently defined as <1% proportion of heavy intensity *Schistosoma* infections) validated in 78 (100%) countries	SAC: 59.3% Adults: 14.3%	Merck KGaA: up to 250 million praziquantel tablets annually for an unlimited period
Soil-transmitted helminthiases (intestinal helminths)	Elimination as a public health problem (<2% proportion of soil-transmitted helminth infections of moderate and heavy intensity due to *Ascaris lumbricoides, Trichuris trichiura, Necator americanus and Ancylostoma duodenal*e) validated in 96 (96%) countries	Pre-SAC: 36.8% SAC: 57.8%	Johnson & Johnson: 200 million mebendazole tablets annually for SAC until 2025 GlaxoSmithKline: 200 million albendazole tablets annually for SAC
Trachoma	Elimination as a public health problem ((i) a prevalence of trachomatous trichiasis ‘unknown to the health system’ of <0.2% in ≥15-year-olds in each formerly endemic district; (ii) a prevalence of trachomatous inflammation—follicular in children aged 1–9 years of <5% in each formerly endemic district; and (iii) written evidence that the health system is able to identify and manage incident cases of trachomatous trichiasis, using defined strategies, with evidence of appropriate financial resources to implement those strategies) validated in 64 (100%) countries	57.2%	Pfizer: unlimited quantity of azithromycin until 2025

Pre-SAC, Pre-school-age children; SAC, School-age children.

These NTDs cause a significant health burden, particularly among the world’s poorest and most marginalised in low-income and middle-income countries.^1 13^ de Vlas *et al*^14^ estimated that if the 2020 goals were achieved for these five NTDs, 328 million disability-adjusted life years (DALYs) would be averted between 2011 and 2030. In addition to their health impact as measured by DALYs, these diseases cause a significant social and economic burden which can exacerbate the cycle of poverty forming an obstacle to sustainable development.^15 16^

WHO and its partners have published a 2021–2030 road map for NTDs^11^ (table 1) which lays out a pathway to sustain the gains and accelerate progress towards the control and elimination of these diseases. Further integration of NTD programmes into local health systems and greater country ownership, including a shift towards increased domestic healthcare spending, will likely be required,^17–19^ and has been set out within the road map as one of three strategic shifts for the coming decade. As the road map calls for country-led, evidence-based planning, there is a need for generation and use of relevant cost and cost-effectiveness data. It is therefore vital that there is a wider understanding of the evidence on the cost-effectiveness of preventive chemotherapy. This will inform future policy decisions and facilitate ongoing provision of resources for programmes.

This paper provides an evaluation of the existing evidence on the cost-effectiveness of the predominantly implemented preventive chemotherapy strategies for lymphatic filariasis, onchocerciasis, schistosomiasis, STH and trachoma. In addition, we highlight areas that require further research.

## Current cost-effectiveness estimates

Cost-effectiveness analyses compare the costs and health effects of an intervention. The morbidities related to these NTDs are complex; infection can lead to different sequelae. Consequently, a variety of different effectiveness metrics have been generated by cost-effectiveness analyses of interventions against these NTDs, including infections averted, disease cases or types of morbidity averted, heavy infections averted and DALYs averted.^20–23^

DALYs are a measure of disease burden and are calculated as the sum of the years of life lost due to premature mortality and the years of healthy life lost due to disability.^24^ One DALY can be interpreted as 1 year of ‘healthy’ life lost.

DALYs averted are a more standardised and comprehensive effectiveness metric than disease cases averted and allow cost-effectiveness estimates to be directly compared between diseases. They are typically the preferred effectiveness metric for interventions in low and lower-middle income countries. Therefore, published literature reporting a cost per DALY averted are the focus of our analysis and we report the estimates related to the preventive chemotherapy strategies predominantly used for these diseases (see box 1). These were assumed to be annual community-wide treatment for lymphatic filariasis, onchocerciasis and trachoma, annual school-based treatment for schistosomiasis and annual or biannual school-based treatment for STH (box 1). Further information regarding cost-effectiveness studies and other economic evaluations relating to these NTDs can be found in previously published reviews.^20–23^ Information on how the literature was identified is provided in box 1.

Box 1 **Selection criteria**

The focus of this analysis was on published estimates reporting a cost per disability-adjusted life year (DALY) averted. We summarised the estimates from published literature reporting average cost-effectiveness ratios related to cost per DALY averted for the predominantly used preventive chemotherapy strategies relative to a do-nothing comparator. The predominantly used strategies were assumed to be annual community-wide treatment for lymphatic filariasis, onchocerciasis and trachoma, annual school-based treatment for schistosomiasis and annual or biannual school-based treatment for soil-transmitted helminthiases (STH). This was informed by the WHO Preventive Chemotherapy and Transmission Control databank (note that for some diseases these are not the only strategies used).^101^ We included estimates related to stand-alone preventive chemotherapy and excluded those in which preventive chemotherapy was combined with additional interventions, such as antibiotic mass drug administration and surgery for trachoma. No time restrictions were made regarding which estimates were included. Cost-effectiveness estimates relating to alternative strategies (as well as incremental cost-effectiveness ratios of scaling up preventive chemotherapy programmes) and estimates relating to treatment in areas below the currently recommended prevalence cut-offs for mass treatment were excluded.^3^ Studies reporting metrics other than a cost per DALY averted were excluded.

We identified the relevant studies from previously published disease-specific systematic reviews^20–23^ and also carried out an updated search for more recent data published in the peer-reviewed press. For trachoma, no previously published systematic review was available and therefore the studies were only identified via the search. Updated searches were of MEDLINE, PubMed and in the reference lists from relevant published articles found within those searches. We used combinations of the search terms ‘lymphatic filariasis’, ‘onchocerciasis’, ‘schistosomiasis’, ‘soil-transmitted helminthiases (STH)’, ‘ascaris’, ‘trichuris’, ‘hookworm’, ‘trachoma’, ‘cost-effectiveness’, ‘cost-utility’, ‘cost per DALY averted’. No language or date restrictions were used. Note: although searches were conducted, this is not a formal systematic review.

It is important to note that we have focused on cost-effectiveness estimates reporting cost per DALY averted relating to the predominantly used preventive chemotherapy strategies for each disease (box 1). However, for some diseases other strategies are also performed (such as treatment of adults for schistosomiasis and treatment of pre-SAC for STH). Estimates also exist relating to alternative strategies, as well as other types of health economic assessments,^20–23 25^ including studies estimating economic and educational benefits.^26^

### How the effectiveness of preventive chemotherapy was quantified

Several methodological factors can influence the effectiveness estimates of preventive chemotherapy, such as the time horizon, the setting under investigation, the modelling approach and the DALY calculation. These need to be considered when comparing different studies (table 2).

**Table 2:** Cost per DALY averted estimates relating to the predominantly used preventive chemotherapy strategies

Study, publication year	Intervention and setting	Approach used to estimate the effectiveness and time horizon	Assumed average costs of preventive chemotherapy	Average cost-effectiveness ratio per DALY averted	Cost year
**Lymphatic filariasis:**					
Remme *et al* (DCP2), 2006^102^	Annual mass community-wide treatment—hypothetical setting (intervention time frame: up to 30 years—depending on when elimination is projected to be achieved)	Back of the envelope (time horizon: 30 years)	Unclear	US$29 within the control scenario and between US$4.40–8.10 within two elimination scenarios	Unclear
Turner *et al*, 2017^57^	Annual mass community-wide treatment—given within GPELF between 2000–2014 (intervention time frame: 15 years)	Static model (time horizon: lifetime of those treated)	Financial cost: US$0.46 per treatment Economic cost (excluding the donated drugs value): US$0.56 per treatment Economic cost (including the donated drugs value): US$1.32 per treatment	Financial cost: US$24 Economic cost (excluding the donated drugs value): US$29 Economic cost (including the donated drugs value): US$64	2014
**Onchocerciasis:**					
Remme *et al* (DCP2), 2006^102^	Annual mass community-wide treatment—given within APOC between 1995–2010 (intervention time frame: 15 years)	Back of the envelope (time horizon: 25 years)	APOC (1995–2010) costing in total US$209 million (financial cost)	US$7	Unclear
Coffeng *et al*, 2013^103^	Annual mass community-wide treatment—given within APOC between 1995–2015 (intervention time frame: 20 years)	Dynamic transmission model (time horizon: 20 years (1995–2015))	Financial delivery cost: US$0.51 per treatment	US$27	Nominal values
Turner *et al*, 2014^104^	Annual mass community-wide treatment—given within a savannah setting in Africa at different levels of endemicity (intervention time frame: up to 50 years—depending on when elimination is projected to be achieved)	Dynamic transmission model (time horizon: 50 years)	Economic delivery cost: US$0.52 per treatment. Drug cost: US$4.21 per treatment	Economic cost (excluding the donated drugs value): US$3–15 Economic cost (including the donated drugs value): US$29–133	2012
**Schistosomiasis:**					
Hotez *et al* (DCP2), 2006^7^	Annual mass school-based treatment—hypothetical setting (intervention time frame: unclear)	Back of the envelope (time horizon: unclear)	Not stated	US$336–692 (note that this at times incorrectly quoted as US$3.36–6.92 within the report^105^	Unclear
GiveWell, 2011^106^	Annual mass school-based treatment—hypothetical setting (intervention time frame: one treatment round)	Back of the envelope (time horizon: one treatment round)	US$0.27–0.47 per treatment (including drug costs)	US$28.19–70.48	Unclear
Lo *et al*, 2016^32^	Annual mass school-based treatment—hypothetical setting (time frame for the intervention: 5 years)	Dynamic transmission model (time horizon: 5 years)	US$0.71 per treatment (including drug costs)	15% prevalence in SAC: US$449 30% prevalence in SAC: US$160	2015
**STH:**					
Chan, 1997^107^	Mass treating SAC against ascaris—within a high prevalence community (intervention time frame: 10 years)	Dynamic transmission model (time horizon: 10 years)	US$1600 to treat the schoolchildren per 100 000 population in China	US$8	Unclear
Miguel and Kremer, 2004^108^	Biannual mass school-based treatment—given within a project in Kenya (intervention time frame: 1 year)	Based on project data (time horizon: 1 year)	Based on US$0.49 per pupil per year (removing the costs related to praziquantel)	US$280 (per STH related DALY averted)	Unclear
Hotez *et al* (DCP2), 2006^7^	Annual mass school-based treatment—hypothetical setting (intervention time frame: unclear)	Back of the envelope (time horizon: unclear)	Not stated	US$326.43 (note that within the report the results were reported as US$3.41 but there were errors within the calculation^105^	Unclear
GiveWell, 2011^106^	Annual mass school-based treatment—hypothetical setting (intervention time frame: one treatment round)	Back of the envelope (time horizon: one treatment round)	US$0.085 per treatment	US$82.54	Unclear
Lo *et al*, 2016^32^	Annual mass school-based treatment—hypothetical setting (intervention time frame: 5 years)	Dynamic transmission model (time horizon: 5 years)	US$0.53 per treatment (including drug costs)	20% prevalence in SAC: US$1077 60% prevalence in SAC: US$298 85% prevalence in SAC: US$174	2015
**Schistosomiasis, lymphatic filariasis and STH:**					
De Neve *et al*, 2018^59^	Annual mass school-based treatment—based on the preventive chemotherapy programme in Madagascar (intervention time frame: one treatment round)	Static model (time horizon: unclear)	Not directly reported	US$125 (95% uncertainty range: 65–231)	2013
**Schistosomiasis and STH:**					
Warren *et al* (DCP1), 1993^109^	Annual mass school-based treatment with an hypothetical setting (intervention time frame: 10 years)	Static calculation (time horizon: 10 years)	US$0.8–1.80 per child per year (including drug costs)	US$6–33	Unclear
Miguel and Kremer, 2004^108^	Annual mass school-based treatment for schistosomiasis and biannual mass school-based treatment for STH—given within a project in Kenya (intervention time frame: 1 year)	Based on project data (time horizon: 1 year)	US$ 0.49 per pupil per year (including drug costs)	US$5 (99% of the benefit was due to averted schistosomiasis)	Unclear
Lo *et al*, 2015^31^	Annual mass school-based treatment—within four communities in Côte d'Ivoire (intervention time frame: 15 years)	Dynamic transmission model (time horizon: 15 years)	US$0.71 per treatment (including drug costs)	US$118 (US$87–140) (92% of the disability resulted from *Schistosoma* infections)	2014

The selection criteria are outlined in Box 1. It was not possible to adjust the different studies for inflation and they are reported in their original cost year.^110^

APOC, African Programme for Onchocerciasis Control; DALY, disability-adjusted life year; DCP1, disease control priorities in developing countries (first edition); DCP2, disease control priorities in developing countries (second edition); GPELF, Global Programme to Eliminate Lymphatic Filariasis; SAC, school-aged children; STH, soil-transmitted helminthiases.

A key consideration is the time horizon of the analyses which determines the duration over which outcomes and costs are calculated (table 2). If the time horizon is too short, the long-term benefits of preventive chemotherapy may not be accounted for, underestimating its cost-effectiveness. As time horizons vary between studies, this parameter is likely a key driver of variability among the estimates. Importantly, many of the studies had time horizons under 10 years and therefore did not fully capture longer-term costs and benefits associated with achieving NTD control or elimination (table 2).

A further important consideration when comparing different studies is the geographical and epidemiological setting being investigated. Some studies focused on a specific area whereas others focused on a whole regional or global programme. The baseline prevalence of infection varied across studies. This impacted effectiveness estimates, because in general, the higher the baseline prevalence, the greater the health impact of preventive chemotherapy and therefore the greater the cost-effectiveness (table 2). Most studies investigated the effect of preventive chemotherapy on one NTD, though a few studies considered the effect on two NTDs and one study considered the simultaneous effect on three NTDs (table 2).

Various approaches have been used in cost-effectiveness studies to quantify the impact of preventive chemotherapy, ranging from back-of-the-envelope calculations to dynamic transmission models (table 2). Dynamic transmission models are often used to estimate the population-level effectiveness of preventive chemotherapy as such models can account for the intervention’s indirect benefits on those not treated and the density-dependent processes which govern infection transmission.^25^ Static models do not account for these processes and therefore may not capture the full benefits of preventive chemotherapy.

DALY calculations are highly sensitive to changes in assigned disability weights and which sequelae of infection are included. Notably, the disability weights used for NTDs have changed significantly since Global Burden of Disease Study (GBD) 2010^27 28^ (see online supplemental Table S1) for the disability weights used by the GBD 2019 for these NTDs). For example, the disability weight for blindness decreased (somewhat controversially) from 0.60 to 0.19.^29 30^ This is an important source of variation in cost-effectiveness estimates and needs to be considered when interpreting available data. DALY calculations for schistosomiasis and STH have been a source of debate and various approaches have been taken in different studies.^23 31–36^ For example, cognitive impairment was removed as a quantifiable sequela of STH infection for GBD 2010. Although this was justified by a perceived lack of evidence of causation,^37^ it is an area of debate within the field.^38 39^ It may be difficult to reach a definitive conclusion regarding the impact of STH on cognition and this should be acknowledged when interpreting cost-effectiveness analyses. Due to the uncertainties associated with the DALY calculations for NTDs, a degree of caution should be employed when interpreting cost-effectiveness estimates, particularly for targeting schistosomiasis and STH in different age groups or in areas of low prevalence and intensity of infection.^40^

10.1136/bmjgh-2021-005456.supp1 Supplementary data

It is important to consider that the framework for estimating DALYs does not necessarily fully summarise the disease burden of these NTDs. As examples, onchocerciasis-associated epilepsy^41 42^ is not fully accounted for, the potential mental health burden for some of the sequelae is not always considered^43 44^ and (due in part to lack of data) potential excess mortality conferred by these diseases is not always included. This could lead to the health impacts being underestimated. Additionally, we recognise that the DALY framework fails to acknowledge the implications of context on the burden of disease, with those living in poverty or with poor access to healthcare typically affected far more than people in higher socioeconomic strata or than those with access to affordable healthcare.^45^

### How costs of preventive chemotherapy were quantified

The way in which costs are quantified will impact the estimates of the cost-effectiveness of preventive chemotherapy. The methods used to parameterise the costs differed widely across studies: some were based on primary data whereas others used assumed crude benchmarks. The delivery costs were generally assumed to be around US$0.50 per treatment (table 2), consistent with current benchmarks.^46^ However, delivery costs vary across different settings with the size of the target population being a key driver in this variation.^20–23 46–50^ This is because delivery costs for preventive chemotherapy tend to show economies of scale that is, as the number of people treated increases, the cost per treatment decreases.^47 51^

It should be noted when looking at the assumed costs that the annual costs of preventive chemotherapy also change depending on the distribution method. The least costly and resource intensive strategy is likely to be incorporating the distribution into an established health system platform/existing programme (such as a Child Health Day), followed by school-based treatment and then community-wide treatment.^21 52^ In addition, the costs are influenced by how the strategy is implemented. For example, the use of volunteer community distributors or teachers would be cheaper than paid health workers.^21 53^

When looking at these studies, it is important to consider whether they are using financial or economic costs (table 2). Financial costs represent the amount paid for the goods, resources and services that are purchased. Economic costs conceptualise costs more broadly and represent the full value of the resources used for an intervention, including the value of donated resources, such as the unpaid time of community health volunteers.^53^ It is typically recommended to use economic costs within economic evaluations.^54 55^ Not all studies clearly reported the type of cost data used or their source (table 2).

As drugs used for these NTDs are typically donated (table 1), they are often not counted as a financial cost for the health ministry within preventive chemotherapy programmes. Their value can, however, be included as an economic cost, depending on the viewpoint from which the intervention’s costs and consequences are evaluated (ie, the study’s perspective). The value of donated medicines can be a significant cost. For example, it has been estimated that the value of pharmaceutical partners’ donated products for the 10 NTDs included in the London Declaration was US$2–3 billion annually.^56^ In practice, it is difficult to estimate the true economic cost of drugs that are donated for preventive chemotherapy programmes, as the assumed costs/value of these drugs varies and the correct value to use is debatable.^20–23 57 58^ If and how the donated drugs are valued are sources of variation in cost-effectiveness estimates of preventive chemotherapy, particularly for ivermectin and azithromycin as the economic values reported by the companies donating them are higher than those for other donated drugs. Such variation needs to be considered when interpreting the results from older costing and cost-effectiveness studies. Furthermore, when a commitment has been made to donate a drug for as long as needed (table 1), it is debatable whether its economic value should be included within economic evaluations.

### Estimates of the cost per DALY averted

Table 2 and box 2 summarise the key cost-effectiveness estimates in terms of DALYs averted relating to the predominantly used preventive chemotherapy strategies. These estimates generally represent the overall mean cost-effectiveness of a NTD preventive chemotherapy programme, or its cost-effectiveness in one specific setting. Importantly, most of the estimates relate to one disease. However, a study by De Neve *et al*^59^ investigated the cost-effectiveness of school-based preventive chemotherapy for multiple diseases (lymphatic filariasis, schistosomiasis and STH), highlighting that the overall cost-effectiveness of preventive chemotherapy depends on how many NTDs treated by the distributed medicines are co-endemic in the location under consideration. Notably, none of the estimates considered the impact of the preventive chemotherapy on non-targeted diseases.

Box 2 **Summary of the cost per disability-adjusted life year (DALY) averted estimates**

**Lymphatic filariasis**: Turner *et al*^57^ estimated that the cost per DALY averted for preventive chemotherapy delivered within the Global Programme to Eliminate Lymphatic Filariasis was US$24 when using financial costs, US$29 when using economic costs excluding the value of the donated drugs and US$64 when using economic costs including the value of the donated drugs (2014 prices). Analysis within the second edition of the disease control priorities in developing countries (DCP2)^102^ estimated that lymphatic filariasis related preventive chemotherapy costs approximately US$29 per DALY averted within a control scenario and between US$4.40–8.10 per DALY averted (*) within two elimination scenarios.

**Onchocerciasis:** Based on dynamic transmission modelling and assuming and economic delivery cost of US$0.52 per treatment, Turner *et al*^104^ estimated that long-term preventive chemotherapy in an African savannah setting cost between US$3–15 per DALY averted (2012 prices) depending on the assumed endemicity level and excluding the value of the donated drugs. The results changed to US$29–133 per DALY averted (2012 prices) when including the additional economic value of the donated ivermectin.^104^ Coffeng *et al*^103^ estimated based on the simulated benefits and reported financial costs of the African Programme for Onchocerciasis Control occurring between 1995–2015, a cost of US$27 per DALY averted (nominal values). Analysis within the DCP2^102^ estimated that onchocerciasis related preventive chemotherapy costs approximately US$7 per DALY averted (*).

**Schistosomiasis:** Lo *et al*^31^ estimated the cost-effectiveness of school-based preventive chemotherapy in four communities in Côte d’Ivoire, resulting in an average cost per DALY averted of US$118 (2014 prices). Most (92%) of the disability resulted from *Schistosoma* infections. Miguel and Kremer^108^ estimated that within their study of school-based preventive chemotherapy in Kenya, it cost US$5 per DALY averted (*) with 99% of the benefit due to averted schistosomiasis. Analysis within the DCP2^7^ estimated that treating school-aged children (SAC) for schistosomiasis costs US$336–692 per DALY averted (*) (note that this is incorrectly quoted as US$3.36–6.92 within the published report).^105^ GiveWell estimated that school-based preventive chemotherapy for schistosomiasis cost US$28.19–70.48 per DALY averted (*).^106^ Lo *et al*^32^ also showed that the cost-effectiveness of school-based preventive chemotherapy against schistosomiasis was highly influenced by the local prevalence of infection—15% prevalence: US$449 per DALY averted, and 30% prevalence: US$160 per DALY averted (2015 prices). Community-wide preventive chemotherapy tended not to be more cost-effective,^23 31 32^ that is, it did not have a lower cost per DALY averted. However, it could be classed as cost-effective depending on the chosen cost-effectiveness threshold.

**Soil-transmitted helminthiases (STH):** Chan^107^ estimated that treating SAC for *Ascaris lumbricoides* is highly cost-effective in a high prevalence community; US$8 per DALY averted (cost year not stated). Analysis within the DCP2^7^ estimated that treating SAC for STH costs US$326 per DALY averted (note that within the report the results were reported as US$3.41 per DALY averted (*), but there were errors within the calculation).^105^ GiveWell re-estimated the cost-effectiveness (using a different methodology) and obtained an estimate of US$83 per DALY averted (*).^105 106^ Miguel and Kremer^108^ estimated that within their study the cost per STH-related DALY averted would be US$280 (*). Lo *et al*^32^ also showed that the cost-effectiveness of school-based preventive chemotherapy against STH was highly influenced by the prevalence of infection—20% prevalence: US$1077 per DALY averted, 60% prevalence: US$298 per DALY averted, 85% prevalence: US$174 per DALY averted (2015 prices).

**Trachoma**: We identified no published cost per DALY averted estimates relating to the currently used strategy of community-wide mass treatment for trachoma using single-dose oral azithromycin. This is now the antibiotic of choice.^111^ In terms of other studies, the Myanmar trachoma control programme was estimated in 1996 by Evans *et al*^112^ to have required, over the course of 30 years implementation, only US$11 for non-surgical interventions (mass treatment with topical antibiotics and community education) for each handicap-adjusted life-year saved. In 2005, Baltussen *et al*^113^ estimated that targeted mass antibiotic treatment of children for trachoma control purposes cost between I$9012–65 022 per DALY averted. (International dollars (I$) are a hypothetical currency unit designed to capture the differences in relative prices across different settings). These estimates included the contemporary market price of azithromycin and its assumed price had a large impact on the results. In practice, virtually all azithromycin used by trachoma programmes is now donated by the manufacturer. When antibiotics are assumed to have no cost to the healthcare system (reflecting donated drugs) or to have been purchased at using generic prices obtainable in India (which are 16 times cheaper), the cost-effectiveness improved, but cost per DALY averted remained relatively high compared with that for other neglected tropical diseases (online supplemental Table S2). In a two-world-region update of the 2005 paper^113^ (again including the cost of azithromycin), Baltussen *et al*,^114^ concluded that targeted mass antibiotic treatment of children with azithromycin to control trachoma could be considered cost-effective in the African Region (I$2101 per DALY averted) but not the South East Asia Region (I$8051 per DALY averted).^114^ Critiques of these analyses have been published.^115 116^ Further analyses are needed before definitive conclusions can be drawn.

Note that it was not possible to adjust the different studies for inflation.*; cost year not clearly stated.

Overall, the cost-effectiveness estimates of preventive chemotherapy against lymphatic filariasis and onchocerciasis that we identified (US$3–133 per DALY averted; table 2) were favourable compared with the main cost-effectiveness thresholds used for low-income countries (outlined in box 3) and compared with other interventions conducted in low-income and middle-income countries, with estimates on par with those associated with interventions for other major global health problems. For example, supplying insecticide-treated nets for malaria has been estimated to cost US$61–94 per DALY averted in three African settings (2012 prices).^60–62^ A comprehensive list of cost-effectiveness estimates for a range of public health interventions is provided by Horton *et al*.^63^

Box 3 **Cost-effectiveness thresholds**

To determine whether an intervention is cost-effective, the cost per disability-adjusted life year (DALY) averted is often compared with a cost-effectiveness (willingness to pay) threshold. The most appropriate cost-effectiveness thresholds to use within global health are under debate.^117–119^ When no country-specific threshold has been set, some studies used the cost-effectiveness thresholds set by the Commission on Macroeconomics and Health^120^; namely a cost per DALY averted <3 or <1 times the country’s gross domestic product (GDP) per capita for an intervention to be considered cost-effective or highly cost-effective, respectively. As a benchmark, using the mean GDP for low-income countries in 2019, these thresholds would be US$2430 and US$810, respectively.^121^ However, these thresholds are now considered too high and have been widely criticised.^65 117–119 122^ The WHO has emphasised that these thresholds were not intended for individual country level-investment decisions but as a broad principle for global or regional consideration. Recent analyses have indicated that a cost-effectiveness threshold of <0.5 times the country’s GDP per capita would be more appropriate for low-income countries^64 65^; corresponding to US$405 based on the average GDP for low-income countries in 2019.^121^ For comparison, the third edition of the Disease Control Priorities project used a threshold of US$200 per DALY averted to identify priority interventions for consideration in low-income countries.^66^ It is vital that conclusions of economic evaluations are re-interpreted in light of such changes: it is likely that some previous conclusions regarding the interventions or strategies that should be rated cost-effective will no longer hold.

The estimated cost-effectiveness of preventive chemotherapy for schistosomiasis and STH was also generally favourable (US$8–1077 per DALY averted; table 2) but more variable than estimates for lymphatic filariasis and onchocerciasis. For schistosomiasis and STH, the estimated cost per DALY averted was generally found to be below a cost-effectiveness threshold less than half the country’s per capita GDP,^64 65^ with some more promising estimates below the disease control priorities (third edition) US$200 per DALY averted threshold.^66^ Here, the highest estimate of US$1077 falls above these conservative thresholds (Box 3) but this is related to a 20% STH prevalence setting below which preventive chemotherapy is not recommended. In addition to the prevalence setting, variation in these estimates is likely to be partly driven by the methods used to calculate corresponding DALY burdens and how they are changing over time. It is debatable whether the number of DALYs averted (which focus on health) are truly capturing all long-term benefits of treating schistosomiasis and STH. Hicks *et al*^67^ recently demonstrated significant long-term economic benefits of deworming children, for example, on household income, and its potential social rate of return.

We identified no published estimates of cost per DALY averted relating to community-wide mass treatment for trachoma. Available cost-effectiveness estimates examined the cost of targeting children only (online supplemental Table S2 and box 1); the currently used strategy is community-wide mass treatment.^68^ Effectiveness trials comparing community-wide treatment to treatment of children only have been conducted^69^ but have not changed global policy. Further health economics analyses are needed before more definitive conclusions can be drawn.

### Implications of these cost-effectiveness estimates

Overall, the reported estimates show that the predominantly used preventive chemotherapy strategies are generally cost-effective, thereby supporting WHO recommendations. The generalisability of these estimates depends on multiple factors, including the epidemiological setting and drivers that influence the delivery costs, such as remoteness and implementation methods. In terms of epidemiological settings, the estimated health benefits and cost-effectiveness of preventive chemotherapy are generally greater for higher transmission settings. Furthermore, the use of volunteer community distributors could have either positive or negative influences on programmatic outcomes depending on the setting.^70^ Hence, it is important to consider these factors when comparing and interpreting different studies for informing policy decisions.

Although the estimates are encouraging, as the financing of NTD programmes shifts towards a greater contribution from endemic countries, it is important that policymakers consider the cost-effectiveness of these interventions relative to other diseases/priorities in their setting. Budget impact analyses may also be required to identify packages that are too expensive.

In terms of priority setting and policy decisions beyond the cost per DALY averted, it is also important to consider the broader socioeconomic benefits of these NTD programmes.^15^ For example, Redekop *et al*^16^ estimated notable social economic benefits would occur from achieving the 2020 targets for these five diseases, both in terms of averted out-of-pocket health expenditure and averted productivity losses (totalling US$229.5 (162.3–344.8) billion in the period 2011–2030 (2015 prices)). Ahuja *et al*^26^ provide a comprehensive summary of the social-economic benefits of deworming. This highlights the broader value of investment in these programmes, particularly in the context of universal health coverage, social protection and reducing inequalities, which are not captured fully by the DALYs averted metric.^15^

## Future research needs

Over the last decade, there has been a notable increase in the number of thorough economic evaluations of preventive chemotherapy, increasing the evidence base for this intervention. However, there are many areas in which further research would help to inform policy.

### Estimating the health benefits of preventive chemotherapy

The current framework for estimating DALYs does not necessarily fully summarise the disease burden of these NTDs, potentially underestimating the cost-effectiveness of interventions against them. Further research is needed to comprehensively capture the health benefits of preventive chemotherapy. For example, how to better estimate the health impacts of preventive chemotherapy (particularly when targeting different age groups) is especially important for schistosomiasis and STH, as well as how to quantify the excess mortality associated with these NTDs. In addition, the potential disease burden associated with mental health issues (such as depression) related to these NTDs is not always currently quantified in the standard DALY calculations for some NTD sequelae. Further investigation is needed of the causal relationship between infection and mental health outcomes and their prevalence—not only for the patients but also their caregivers.^43 71^

In future studies, there is a vital need for greater transparency on how numbers of DALYs averted are estimated, particularly with respect to disability weights and their source. Any changes from the approach used within the most recent GBD study needs to be clearly stated and justified.

Cost-effectiveness analyses published to date have typically investigated the impact of preventive chemotherapy on one NTD (table 2). However, preventive chemotherapy for NTDs uses relatively broad-spectrum drugs that can also have an impact on other co-endemic infections,^72–77^ or even on all-cause morbidity and mortality.^78–80^ For example, mass ivermectin distribution will also have an impact on strongyloidiasis, scabies, ectoparasites and mosquito mortality.^73 75 81–84^ Consequently, the overall cost-effectiveness of preventive chemotherapy is likely to be underestimated. Further quantification of these auxiliary benefits is needed.

Beyond this, it is important that further research is conducted on the broader non-health-related benefits of preventive chemotherapy, including socioeconomic benefits such as educational and occupational outcomes, productivity gains, and financial protection.

### Integrated NTD control

As NTD programmes push towards greater integration (whereby multiple disease-specific programmes are delivered within a single programme or local health system) and expand their footprint towards the provision of universal health coverage, it would be useful for decision-makers to be able to consider cost-effectiveness of integrated NTD control programme packages, rather than stand-alone disease-specific interventions.^17–19^ A better understanding of the costs of integrated control programmes^85 86^ and how integration may influence the costs and cost-effectiveness of implementing different control strategies will be required for this.

Further economic evaluations on areas where NTDs are co-endemic are important as a study by Lo *et al*^32^ highlighted that the optimal strategy for schistosomiasis and STH can depend on whether they are co-endemic.

### Economic evaluations of alternative preventative chemotherapy strategies and complementary interventions

A key research area for these NTDs is where and when alternative preventive chemotherapy strategies should be used, such as increasing treatment frequency, targeting different age groups or using a different drug or drug combination. There is a need for more data on the relative costs of different preventive chemotherapy strategies in different settings. For example, there are few primary data on the relative cost of school-based versus community-wide preventive chemotherapy.^21^ Concerningly, many economic evaluations on this topic make assumptions regarding the relative cost of these strategies based on limited data. This is a research gap that needs to be addressed to inform policy. Due to economies of scale and variation in delivery costs between areas, it is usually not possible to generalise cost data from different studies for this purpose.^47^

The potential for leveraging existing delivery platforms, such as child health days or antenatal clinics, could be considered more often when evaluating possible preventive chemotherapy strategies. It is likely that building on established health system platforms to deliver treatment could be cheaper than undertaking dedicated preventive chemotherapy.^17 87^ For example, Boselli *et al*^88^ estimated that adding deworming into an existing immunisation and vitamin A supplementation campaign would cost less than US$0.01 per deworming treatment. Further implementation research is needed to assess the possible use of such platforms,^89^ with potential benefits and risks evaluated. This may be particularly important when considering a shift from school-based to community-wide treatment for schistosomiasis and STH. If sufficient coverage of high-risk adults for schistosomiasis and STH could be achieved with established platforms, using them could be more cost-effective than community-wide mass treatment.

More economic evaluations on complementary interventions for these NTDs, such as water, sanitation and hygiene measures, behaviour change interventions and vector control are required. In addition to preventive strategies, it is also important to further evaluate the cost-effectiveness of morbidity management strategies.

Further research is required for settings where onchocerciasis is co-endemic with loiasis. Individuals heavily infected with loiasis can experience severe and sometimes fatal neurological sequelae after ivermectin treatment.^90 91^ Ivermectin-based mass drug administration is not recommended in hypo-endemic areas for onchocerciasis (<40% microfilariae or <20% nodules prevalence) that are co-endemic for loiasis.^92^ Consequently, alternative preventive chemotherapy strategies are needed in these settings. One solution is screening every member of the population for loiasis before treatment, treating all those with no or low-intensity *Loa loa* infection and excluding those with a high-intensity infection. However, such a test-and-(not)-treat approach is more costly than standard preventive chemotherapy.^93^ Lymphatic filariasis control strategies using ivermectin are also impeded in settings co-endemic with loiasis and need to be adapted. In these areas, twice-yearly albendazole monotherapy in combination with coordinated vector control is the recommended strategy.^94^ This strategy will require additional cost due to additional rounds of treatment compared with multi-drug combinations. There is a need for further economic evaluations of the optimal alternative strategies for these settings. Importantly, these will need to consider not only the costs and benefits but also consider the programme goals as a whole and the risk of such settings acting as sources of re-infection to neighbouring populations.

When considering the cost-effectiveness of new strategies or interventions, an important consideration is whether the goal is disease/morbidity control or interruption of transmission.^20 22^ Which strategies are cost-effective can be influenced by the goal of the programme. More intensive strategies may not be cost-effective when the goal is controlling morbidity but could be when aiming for interruption of transmission.^20 22^ For example, though complementary vector control for lymphatic filariasis has a notable effect on transmission,^95 96^ it may not necessarily be cost-effective for controlling morbidity (ie, in terms of the additional health benefits gained from that setting compared with preventive chemotherapy only) but could be cost-effective in terms of its long-term impact on transmission in the context of achieving elimination goals (such as reducing the risk of resurgence).^20^ The framework for the economic evaluation of elimination strategies needs further investigation and development. In particular, a pressing need is how we evaluate the cost-effectiveness of maintaining control versus moving towards interruption of transmission and the cost-effectiveness of adding complementary interventions (such as a combined preventive chemotherapy and vector control strategy).

### Contextual factors that affect the cost-effectiveness of NTD programmes

Current cost-effectiveness estimates generally represent the overall mean cost-effectiveness of a NTD preventive chemotherapy programme, or its cost-effectiveness in one specific setting. Further investigation is needed of the contextual factors that affect cost-effectiveness estimates and their potential variation across different settings.

A key contextual factor is the delivery cost of preventive chemotherapy. There is a need for further cost data from a wider range of settings and programmatic contexts.^46^ These delivery costs may be influenced by the stability and accessibility of the area (such as access to forest settings) and the costs associated with expanding treatment programmes to target harder-to-reach areas/groups may have diseconomies of scale.^47^ Due to this, the cost per treatment of NTD control programmes will likely increase as end goals are approached.^47^

The epidemiological setting, such as the level of transmission (baseline prevalence of infection), endemic NTD species, age profile of infected individuals and historic coverage, can notably influence the cost-effectiveness of different preventive chemotherapy strategies. For example, the benefits of moving from school-based to community-wide treatment for schistosomiasis and STH depends on the burden of infection in adults.^21 23 40 97^ The impact of the epidemiological setting on the generalisability of studies needs further investigation, particularly when considering a change in strategy. If such variation in epidemiological settings is not properly accounted for there is a danger that studies will be overgeneralised leading to inappropriate policy decisions.

## Conclusion

Many studies have found preventive chemotherapy to be a cost-effective strategy for controlling several NTDs. The cost per DALY averted estimates relating to community-wide preventive chemotherapy for lymphatic filariasis and onchocerciasis were particularly favourable when other public health interventions. The estimates for school-based preventive chemotherapy for schistosomiasis and STH were also generally favourable but more variable. There were no estimates of cost per DALY averted relating to community-wide mass antibiotic treatment for trachoma. It is important to acknowledge that the DALYs averted metric may not be capturing the broader socioeconomic benefits for controlling these diseases.

Overall, these findings support the need for continuing NTD control and elimination efforts, providing important implications for advocacy groups, potential funders and decision-makers in endemic countries to justify the increased domestic healthcare spending needed for NTD programmes. These findings and the need for further work are increasingly important in light of the current coronavirus 2019 (COVID-19) pandemic, as there is a risk that there will be even more limited resources and financing to resume public health programmes in the coming years.^98 99^

Although these results are promising, it does not mean that preventive chemotherapy is a cost-effective intervention in every setting where these infections are endemic. Hence, WHO recommendations on use of different strategies depending on the endemicity of these diseases (including, at times a minimum infection prevalence threshold for mass treatment) is underlined. Accordingly, there is no one-size-fits-all approach regarding the best preventive chemotherapy strategy for controlling NTDs as this will depend on the local setting: which NTDs are endemic, at what levels of transmission, and whether the goal is controlling morbidity or interruption of transmission. Further work including cost-effectiveness analyses is needed to inform preventive chemotherapy programmes and the economics of elimination programmes.

## Data Availability

All data relevant to the study are included in the article.
