# Supplementary material for: Are current preventive chemotherapy strategies for controlling and eliminating neglected tropical diseases cost-effective?
Source: BMJ Glob Health. 2021 Aug 12;6(8):e005456. doi: 10.1136/bmjgh-2021-005456 (PMC8362715; doi:10.1136/bmjgh-2021-005456)
Supplement: Supplementary data [file bmjgh-2021-005456supp001.pdf]

Turner *et al.*, Are current preventive chemotherapy strategies for controlling and eliminating neglected tropical diseases cost-effective?

Supporting Table S1: Global Burden of Disease Study 2019 (GBD 2019) Disability Weights [1]

Disease	Sequela	Disability weight
**Lymphatic filariasis**		
Hydrocele due to lymphatic filariasis	Epididymo-orchitis	0.128 (0.086-0.180)
Lymphedema due to lymphatic filariasis	Lymphatic filariasis, symptomatic	0.109 (0.073-0.154)
Acute adenolymphangitis due to lymphatic filariasis	Infectious disease, acute episode, moderate	0.051 (0.032-0.074)
**Onchocerciasis**		
Mild skin disease without itch due to onchocerciasis	Disfigurement, level 1	0.011 (0.005-0.021)
Mild skin disease due to onchocerciasis	Disfigurement, level 1 with itch/pain	0.027 (0.015-0.042)
Moderate skin disease due to onchocerciasis	Disfigurement, level 2, with itch/pain	0.188 (0.125-0.267)
Severe skin disease due to onchocerciasis	Disfigurement, level 2, with itch/pain	0.188 (0.125-0.267)
Severe skin disease without itch due to onchocerciasis	Disfigurement, level 3	0.405 (0.275-0.546)
Moderate vision impairment due to onchocerciasis	Distance vision, moderate impairment	0.031 (0.019-0.049)
Severe vision impairment due to onchocerciasis	Distance vision, severe impairment	0.184 (0.125-0.258)
Blindness due to onchocerciasis	Distance vision blindness	0.187 (0.124-0.260)
**Schistosomiasis**		
Mild schistosomiasis	Infectious disease, acute episode, mild	0.006 (0.002-0.012)
Mild anemia due to schistosomiasis	Anemia, mild	0.004 (0.001-0.008)
Moderate anemia due to schistosomiasis	Anemia, moderate	0.052 (0.034-0.076)
Severe anemia due to schistosomiasis	Anemia, severe	0.149 (0.101-0.209)
Mild diarrhea due to schistosomiasis	Diarrhea, mild	0.074 (0.049-0.104)
Dysuria due to schistosomiasis	Abdominopelvic problem, mild	0.011 (0.005-0.021)
Bladder pathology due to schistosomiasis	Abdominopelvic problem, mild	0.011 (0.005-0.021)
Hydronephrosis due to schistosomiasis	Abdominopelvic problem, mild	0.011 (0.005-0.021)
Hepatomegaly due to schistosomiasis	Abdominopelvic problem, mild	0.011 (0.005-0.021)
Ascites due to schistosomiasis	Abdominopelvic problem, moderate	0.114 (0.078-0.159)
Hematemesis due to schistosomiasis	Gastric bleeding	0.325 (0.209-0.462)
**Soil-transmitted helminthiases**		
Heavy infestation of ascariasis	Intestinal nematode infections, symptomatic	0.027 (0.015-0.043)
Mild abdominopelvic problems due to ascariasis	Abdominopelvic problem, mild	0.011 (0.005-0.021)
Severe wasting due to ascariasis	Severe wasting	0.128 (0.082-0.183)
Heavy infestation of trichuriasis	Intestinal nematode infections, symptomatic	0.027 (0.015-0.043)
Mild abdominopelvic problems due to trichuriasis	Abdominopelvic problem, mild	0.011 (0.005-0.021)
Severe wasting due to trichuriasis	Severe wasting	0.128 (0.082-0.183)
Heavy infestation of hookworm	Intestinal nematode infections, symptomatic	0.027 (0.015-0.043)
Mild abdominopelvic problems due to hookworm disease	Abdominopelvic problem, mild	0.011 (0.005-0.021)
Severe wasting due to hookworm disease	Severe wasting	0.128 (0.082-0.183)
Mild anemia due to hookworm disease	Anemia, mild	0.004 (0.001-0.008)
Moderate anemia due to hookworm disease	Anemia, moderate	0.052 (0.034-0.076)
Severe anemia due to hookworm disease	Anemia, severe	0.149 (0.101-0.209)
**Trachoma**		
Moderate vision impairment due to trachoma	Distance vision, moderate impairment	0.031 (0.019-0.049)
Severe vision impairment due to trachoma	Distance vision, severe impairment	0.184 (0.125-0.258)
Blindness due to trachoma	Distance vision blindness	0.187 (0.124-0.260)

Turner *et al.*, Are current preventive chemotherapy strategies for controlling and eliminating neglected tropical diseases cost-effective?

Supporting Table S2: The cost per disability-adjusted life year (DALY) averted estimates relating to the preventive chemotherapy for trachoma.

Study	Intervention and setting	Approach used to estimate the effectiveness and time horizon	Assumed average costs of preventive chemotherapy	Average cost-effectiveness ratio	Cost year
Baltussen *et al.* [2]	Antibiotic treatment of all children (aged 1–10 years) for ten years - 7 regions	Static model (time horizon: lifetime of those treated)	Cost per patient I$13.25 (African Region D): including azithromycin at prevailing market prices.	Cost-effectiveness ranged between I$9,012 and I$65,022 per DALY averted.<br><br>For the African Region D, the results changed from I$9,012 to I$3,922 per DALY averted when assuming the drug was donated.	2000 prices
Baltussen *et al.* [3]	Antibiotic treatment of all children (aged 1–10 years) for ten years - 2 regions	Static model (time horizon: lifetime of those treated)	Annual cost per capita:<br>African Region E: I$0.28<br>South-East Asian Region D: I$0.20 (including the price of azithromycin)	African Region E: I$2,101 per DALY averted<br><br>South-East Asian Region D: I$8,051 per DALY averted	2005 prices

It should be noted that the cost-effectiveness estimates for combined surgery and preventive chemotherapy were more promising than just stand-alone preventive chemotherapy.
I$: International dollar (a hypothetical currency unit that is designed to capture the differences in relative prices across different settings. For example, I$1 would buy in the country of interest a comparable amount of goods and services as US$1 in the United States).

Turner *et al*., Are current preventive chemotherapy strategies for controlling and eliminating neglected tropical diseases cost-effective?

References

1. Global Burden of Disease Study 2019 (GBD 2019) Data Resources. http://ghdx.healthdata.org/gbd-2019.
2. Baltussen RM, Sylla M, Frick KD, Mariotti SP. Cost-effectiveness of trachoma control in seven world regions. *Ophthalmic epidemiology* 2005; **12**(2): 91-101.
3. Baltussen R, Smith A. Cost effectiveness of strategies to combat vision and hearing loss in sub-Saharan Africa and South East Asia: mathematical modelling study. *BMJ (Clinical research ed)* 2012; **344**: e615-e.
